# Ultra-Low-Dose Whole-Body Computed Tomography Protocol Optimization for Patients With Plasma Cell Disorders: Diagnostic Accuracy and Effective Dose Analysis From a Reference Center

**DOI:** 10.3389/fonc.2021.769295

**Published:** 2021-11-17

**Authors:** Davide Tore, Osvaldo Rampado, Carla Guarnaccia, Roberto Mina, Maria Oronzio, Ambra Santonocito, Alessandro Serafini, Giulio Antonino Strazzarino, Laura Gianusso, Sara Bringhen, Paolo Fonio, Alessandro Depaoli

**Affiliations:** ^1^ Radiology Unit, Department of Surgical Sciences, University of Turin, Azienda Ospedaliero Universitaria (A.O.U.) Città della Salute e della Scienza di Torino, Turin, Italy; ^2^ Medical Physics Unit, Azienda Ospedaliero Universitaria (A.O.U.) Città della Salute e della Scienza di Torino, Turin, Italy; ^3^ Myeloma Unit, Division of Hematology, University of Turin, Azienda Ospedaliero Universitaria (A.O.U.) Città della Salute e della Scienza di Torino, Turin, Italy; ^4^ Oncohematology and Multiple Myeloma Clinical Trials Unit, Department of Oncology, University of Turin, Azienda Ospedaliero Universitaria (A.O.U.) Città della Salute e della Scienza di Torino, Turin, Italy

**Keywords:** ultra-low-dose whole-body CT, multiple myeloma, plasma cell disorders, effective dose, dose reduction

## Abstract

**Background:**

The whole-body low-dose CT (WBLDCT) is the first-choice imaging technique in patients with suspected plasma cell disorder to assess the presence of osteolytic lesions. We investigated the performances of an optimized protocol, evaluating diagnostic accuracy and effective patient dose reduction using a latest generation scanner.

**Methods and Materials:**

Retrospective study on 212 patients with plasma cell disorders performed on a 256-row CT scanner. First, WBLDCT examinations were performed using a reference protocol with acquisition parameters obtained from literature. A phantom study was performed for protocol optimization for subsequent exams to minimize dose while maintaining optimal diagnostic accuracy. Images were analyzed by three readers to evaluate image quality and to detect lesions. Effective doses (E) were evaluated for each patient considering the patient dimensions and the tube current modulation.

**Results:**

A similar, very good image quality was observed for both protocols by all readers with a good agreement at repeated measures ANOVA test (p>0.05). An excellent inter-rater agreement for lesion detection was achieved obtaining high values of Fleiss’ kappa for all the districts considered (p<0.001). The optimized protocol resulted in a 56% reduction of median DLP (151) mGycm, interquartile range (IQR) 128–188 mGycm vs. 345 mGycm, IQR 302–408 mGycm), of 60% of CTDIvol (2.2 mGy, IQR 1.9–2.7 mGy vs. 0.9 mGy, IQR 0.8–1.2 mGy). The median E value was about 2.6 mSv (IQR 1.7–3.5 mSv) for standard protocol and about 1.5 mSv (IQR 1.4–1.7 mSv) for the optimized one. Dose reduction was statistically significant with p<0.001.

**Conclusions:**

Protocol optimization makes ultra-low-dose WBLDCT feasible on latest generation CT scanners for patients with plasma cell disorders with effective doses inferior to conventional skeletal survey while maintaining excellent image quality and diagnostic accuracy. Dose reduction is crucial in such patients, as they are likely to undergo multiple whole-body CT scans during follow-up.

## Introduction

Multiple myeloma (MM) is the second most common hematological malignancy, characterized by bone marrow infiltration by monoclonal plasma cells that ultimately leads to end-organ damage (1). Monoclonal gammopathy of undetermined significance (MGUS) is a premalignant plasma cell disorder preceding MM, with a risk of progression to MM of about 1% per year. Smoldering MM (SMM) represents an intermediate clinical condition between MGUS and MM, characterized by a bone marrow plasma cell infiltration ≥10% without signs or symptoms related to MM itself, with a risk of progression to symptomatic myeloma of about 10% per year (1).

The diagnosis of MM, based on the International Myeloma Working Group (IMWG) criteria, requires a bone marrow plasma cell infiltration ≥10% and at least one CRAB syndrome feature (hypercalcemia, renal failure, anemia, and bone lesions), or ≥60% monoclonal plasma cell infiltration on bone marrow biopsy, serum involved to uninvolved free light chain ratio ≥100 or more than one >5 mm focal lesion at magnetic resonance imaging or a biopsy-proven bone or extramedullary plasmacytoma.

Bone disease is the most frequent symptom related to MM; up to 80% of patients with newly diagnosed MM have osteolytic lesions (2, 3), and about 90% of patients develop bone lesions throughout the course of their disease (4).

Imaging plays a crucial role in plasma cell disorders for the diagnosis, monitoring, and management of the disease. Conventional skeletal survey (CSS) has been replaced by more advanced and sensible imaging modalities (5–7) such as whole-body MRI (WBMRI), whole-body low-dose CT (WBLDCT), or FDG/PET-CT (8–11). WBLDCT currently represents the first-choice imaging technique in patients with a suspected plasma-cell disorder to assess the presence and the extension of osteolytic lesions (12). WBLDCT has several advantages over WBMRI, as it is widely available, cheap, simple to perform, and well tolerated by patients due to a very short scan time.

Since the introduction of CT for bone evaluation in patients with MM and other plasma cell disorders, many efforts were implemented to reduce radiation exposure. As such population is likely to undergo multiple whole-body exams during treatment (13) and follow-up, it is of the utmost importance to reduce radiation dose, as performing exams with standard-dose protocol would cause unacceptably high cumulative exposures.

The aim of this study was to investigate the performances of a whole-body ultra-low-dose CT protocol, evaluating diagnostic accuracy and effective patient dose reduction achievable through a latest generation CT scanner.

## Materials and Methods

### Phantom Study and Protocol Optimization

The study was performed using a 256-slice CT scanner (Revolution CT, GE, USA). First, WBLDCT examinations in our department were performed in 2018 starting from a reference protocol with acquisition parameters set considering literature indications (14) (120 kV; collimation, 80 mm; rotation time, 0.28 s; pitch, 0.9; slice thickness, 1.25 mm; noise index (NI), 25; and iterative, ASIR-V 50%).

In February 2019, a phantom study was conducted to investigate the minimum dose that allowed to maintain an optimal diagnostic accuracy in lesion detection (15).

Starting from the reference protocol, the phantom was repeatedly scanned varying several acquisition parameters. In particular, in a first measurement session, the impact of different levels of iterative reconstruction ASIR-V (50%, 70%, 80%, and 100%) combined with two noise index values (25 and 50) and different kilovoltages (100, 120, and 140 kV) was investigated and used to identify the best ASIR-V percentage.

A second measurement session was performed varying kilovoltages (100, 120, and 140 kV) and the noise index (21, 25, 28, 35, and 48) in order to obtain a CTDI vol in a range from 0.3 to 1.4 mGy.

A noise analysis was performed using an automatic multiple region of interest (ROI) evaluation made by a homemade tool (implemented as a plugin of the software ImageJ, https://imagej.nih.gov/ij/index.html). Each slice was subdivided in square ROIs of 10 per 10 voxels. Objective image noise was calculated as the standard deviation of the pixel values for each ROI that was located entirely within a soft-tissue-simulating material. The median noise value was calculated for each image and compared with the set noise index, along all the phantom length. The median noise slice by slice trend and the tube current modulation trend (obtained extracting current values from the images DICOM header) were plotted over the z-axis.

The noise power spectrum in homogeneous regions and the bone edge profiles were analyzed for some images.

Each data set was scored individually by two senior radiologists (AD and CG) and a resident (DT) using a Likert scale (from 1, non-diagnostic to 3, average still diagnostic to 5, optimal), evaluating four separate anatomical areas: skull base, thoracic spine, pelvis, and distal femora.

### Effective Dose Evaluation

All CT acquisition parameters and dose indexes [CTDI_vol_ and dose length product (DLP)] were collected by a dose registration system (Physico, Emme Esse, Italy) integrated with a software for CT effective dose (E) estimation (Virtual Phantom CT). For each patient, a virtual phantom with the correspondent gender and body size was selected among the available options, considering the patient body mass index (BMI) with the following thresholds: ≤25 kg m^−2^ normal weight adult, >25 and ≤30 kg m^−2^ overweight adult, >30 and ≤ 35 kg m^−2^ obese level I adult, >35 and ≤40 kg m^−2^ obese level II adult, and >40 kg m^−2^ morbidly obese adult.

**Figure 1:**
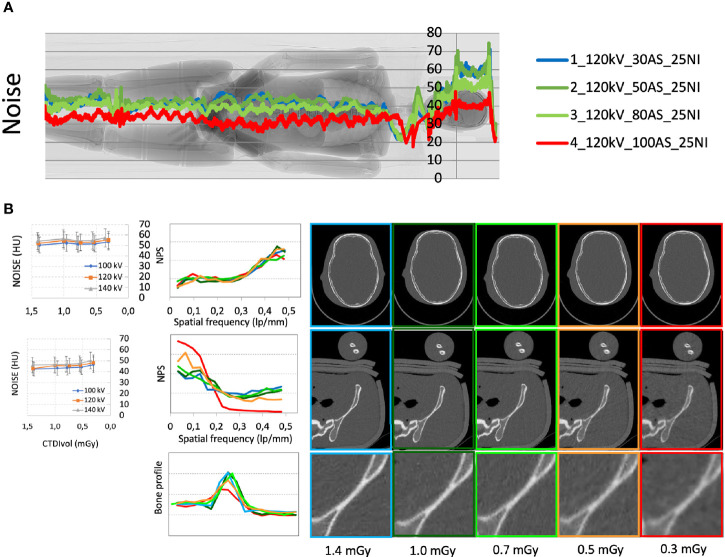
**(A)** Median noise trend with different acquisition parameters superimposed to the phantom scout image. **(B)** Noise (expressed in HU) and noise power spectrum (NPS) for the skull and pelvis districts calculated with different acquisition parameters and doses; detail of the bone edge profile in the pelvis with different acquisition parameters.

**Figure 1** shows an example of tube current modulation trend along the patient length. Tube current values were at the minimum settable of 10 mA over the patient head and lower limbs, whereas typical values in the range of 50–100 mA were observed for the patient chest and abdomen. To account of this dose modulation, the E for each patient was calculated by Virtual Phantom CT considering the relative contribution to average CTDI_vol_ and total DLP of each anatomical district (head, neck, chest, abdomen, pelvis, and lower limbs). The contributions for each district were summed to obtain the total E.

### *In Vivo* Validation

The study, approved by the Ethics Committee of our Institution, was conducted on consecutive patients who underwent WBLDCT at our department between 2018 and 2021 for diagnostic workup of plasma cell disorders, divided in two groups depending on the scanning protocol: group 1 standard protocol (23 patients, 10 women) and group 2 low-dose protocol (189 patients, 84 women). Complete patient characteristics are reported in **Table 1**.

**Table 1:** Characteristics of the patients.

Characteristics of the patients		
	Group 1 standard protocol	Group 2 low-dose protocol
Number	23	189
Sex	10 female 13 male	84 female 105 male
Age (years)	66.74 ± 2.51	68.94 ± 0.80
Number of patients by age:		
• <40 years	0 (0%)	1 (0,5%)
• 40–50 years	3 (13%)	11 (5,8%)
• 50–60 years	4 (17.4%)	28 (13.2%)
• 60–70 years	5 (21.7%)	47 (24.9%)
• 70–80 years	8 (34.7%)	68 (36%)
• 80–90 years	3 (13%)	33 (17.4%)
• Over 90 years	0 (0%)	1 (0.5%)
BMI (kg m^−2^)	25.6 ± 5.7	25.1 ± 5.4
Plasma cell disorder:		
• Multiple myeloma	12 (52.1%)	85 (44.9%)
• Smoldering myeloma	1 (4.3%)	20 (10.6%)
• MGUS	9 (39.1%)	82 (43.4%)
• Plasmacytoma	0 (0%)	1 (0.5%)
• Plasma cell leukemia	1 (4.3%)	0 (0%)
• Other	0 (0%)	1 (0.5%)

Each data set was evaluated for image quality by three radiology residents (ASa, ASe and GS) proficient in oncologic imaging with the Likert scale used for the phantom study (from 1, non-diagnostic to 3, average still diagnostic to 5, optimal) considering four anatomic districts: skull base, thoracic spine, pelvis, and distal femora.

WBLDCT exams were independently analyzed by the three readers looking for cortical lytic bone lesions and extraosseous localizations, reported for the following anatomic districts: skull, cervical spine, thoracic spine, lumbar spine, humeral head, humeral diaphysis, scapula, clavicula, coxae-sacrum, femoral head, femoral diaphysis, sternum, ribs, and non-osseous localizations.

Cortical lytic lesions were assessed with a Likert scale from 1 to 5 depending on diagnostic confidence (1, definite non-osteolysis; 2, probably non-osteolysis; 3, probable osteolysis; 4, highly suspicious for osteolysis; 5, definite osteolysis) and reported for the same districts considered for lesion detection.

### Statistics

Statistical analysis was performed with open-source software jamovi [The jamovi project (2020); jamovi (Version 1.6.14.0) (Computer Software), retrieved from https://www.jamovi.org].

Continuous variables were expressed as mean ± standard deviation (SD); CTDI_vol_, DLP, and E were also expressed as median and interquartile ranges (IQR) to minimize the effect of extreme values.

Discrete variables were expressed as absolute numbers and percentages.

Statistical tests Student’s t, repeated measures ANOVA, and Fleiss’ kappa were used when appropriate.

A p<0.05 was considered statistically significant.

## Results

### Phantom Study Results

In the first measurement session, it was stated that the optimal setting for ASIR-V iterative reconstruction was 80%, regardless the other acquisition parameters.

In the second measurement session by consensus of two radiologists, it was established that the optimal combination of acquisition parameters to obtain the best achievable image quality with minimum dose of 120 kV with automatic mAs modulation, ASIR-V of 80%, and NI of 28 resulting in an average computed tomography dose index (CTDI_vol_) of 0.7 mGy for the phantom WBLDCT. With such acquisition setting, images received a Likert score of 5 by both readers for all districts considered.

The median noise trend with different acquisition parameters is plotted in **Figure 1A** superimposed to the phantom scout image.

The noise quantified with multiple automatic ROIs with different acquisition parameters is shown in **Figure 1B** for the skull and pelvis.

The noise power spectrum (NPS) of the different acquisitions for the skull and pelvis districts is reported in **Figure 1B**. NPS at different kVs and doses presented no statistically significant differences in the skull district (p>0.05), whereas a different distribution of NPS was observed in particular in the pelvis district for CTDI_vol_ values below 0.7 mGy. These lower-dose acquisitions also highlighted a degradation of the analyzed bone edge profiles. In summary, the quantitative analysis was consistent with the subjective image evaluation and supported the choice of a NI level of 28.

The new optimized protocol was compliant with the recommendations of the IMWG bone working group, with thin slice acquisition (1.25 mm); images were acquired from the cranial vault to proximal tibial metaphysis including humeri in the field of view and reconstructed with sharp (bone, slice thickness, 0.625 mm) and standard algorithm (12).

This low-dose protocol was implemented in clinical practice since April 2019.

### Effective Dose Evaluation

**Figure 2:**
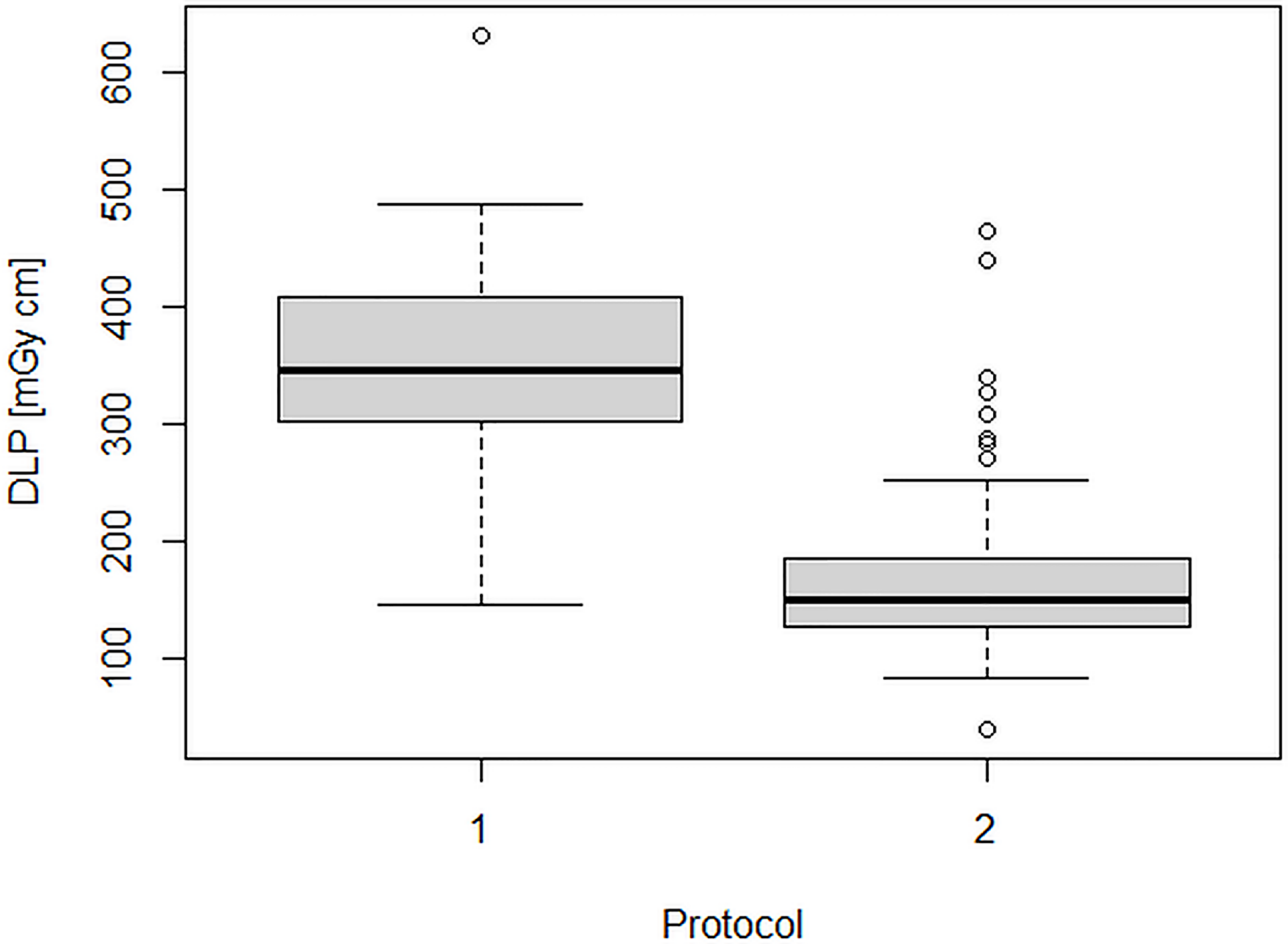
DLP distributions for the original protocol (1) and for the optimized protocol (2).

**Figure 3:**
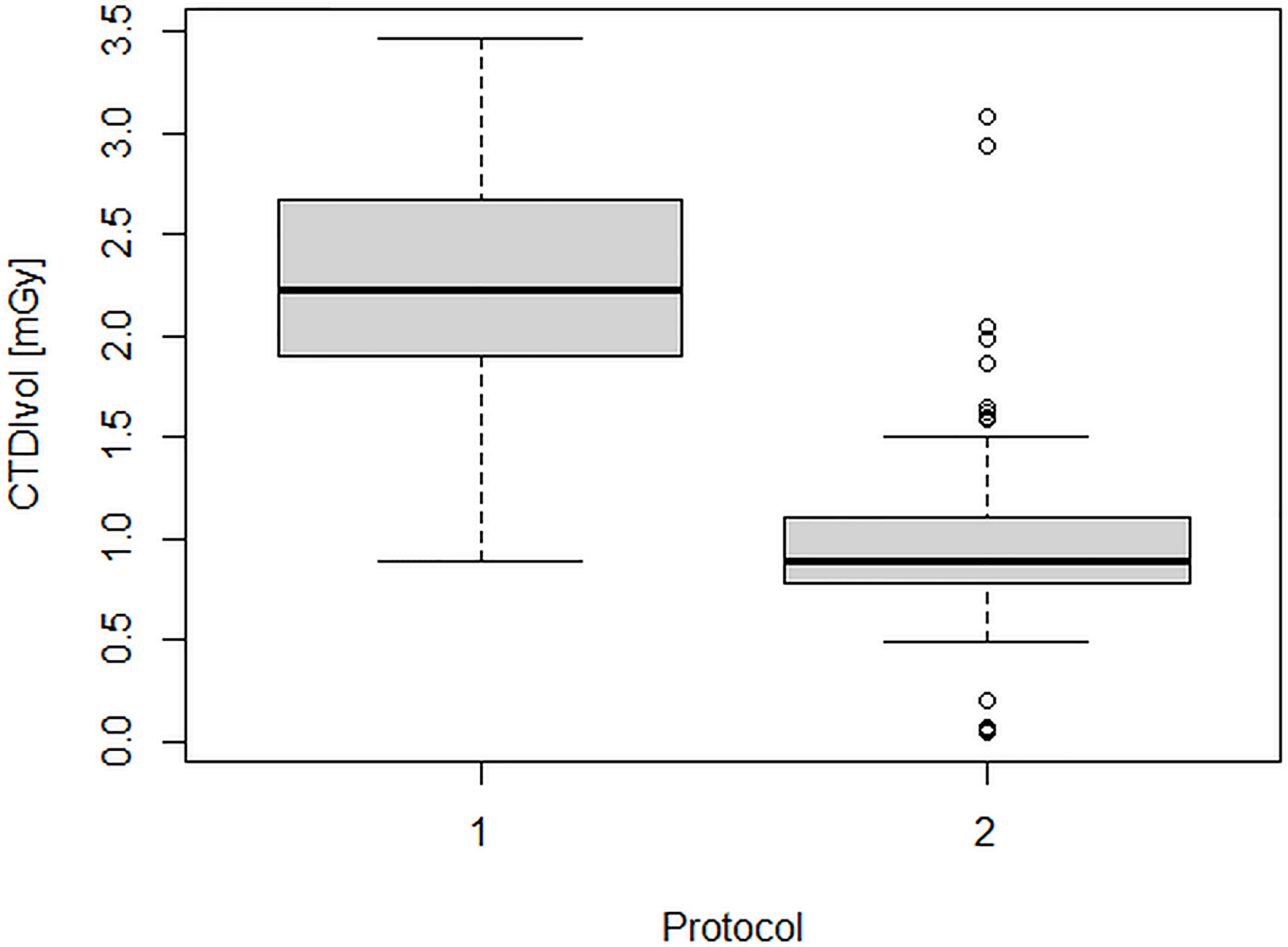
CTDI distributions for the original protocol (1) and for the optimized protocol (2).

**Figures 2** and **3** show the dose indicators DLP and CTDI distributions for the original protocol and for the optimized protocol. DLP median value was 345 mGycm for the original protocol (mean value, 342.5 ± 102 mGycm), with an interquartile range (IQR) of 302–408 mGy cm. With the optimized protocol, a 56% reduction of the median DLP was observed, resulting in a value of 151 mGy cm (IQR 128–188 mGy cm, mean 160.5 ± 55 mGy cm). A dose reduction of about 60% was observed also for CTDI_vol_, from an initial median value of 2.2 mGy (IQR 1.9–2.7 mGy, mean 2.2 ± 0.6 mGy cm) down to a median CTDIvol of 0.9 mGy (IQR 0.8–1.2 mGy, mean 1 ± 0.4 mGy cm). Dose reduction was statistically significant for both DLP and CTDI_vol_ (p<0.001 in both cases).

**Figure 4:**
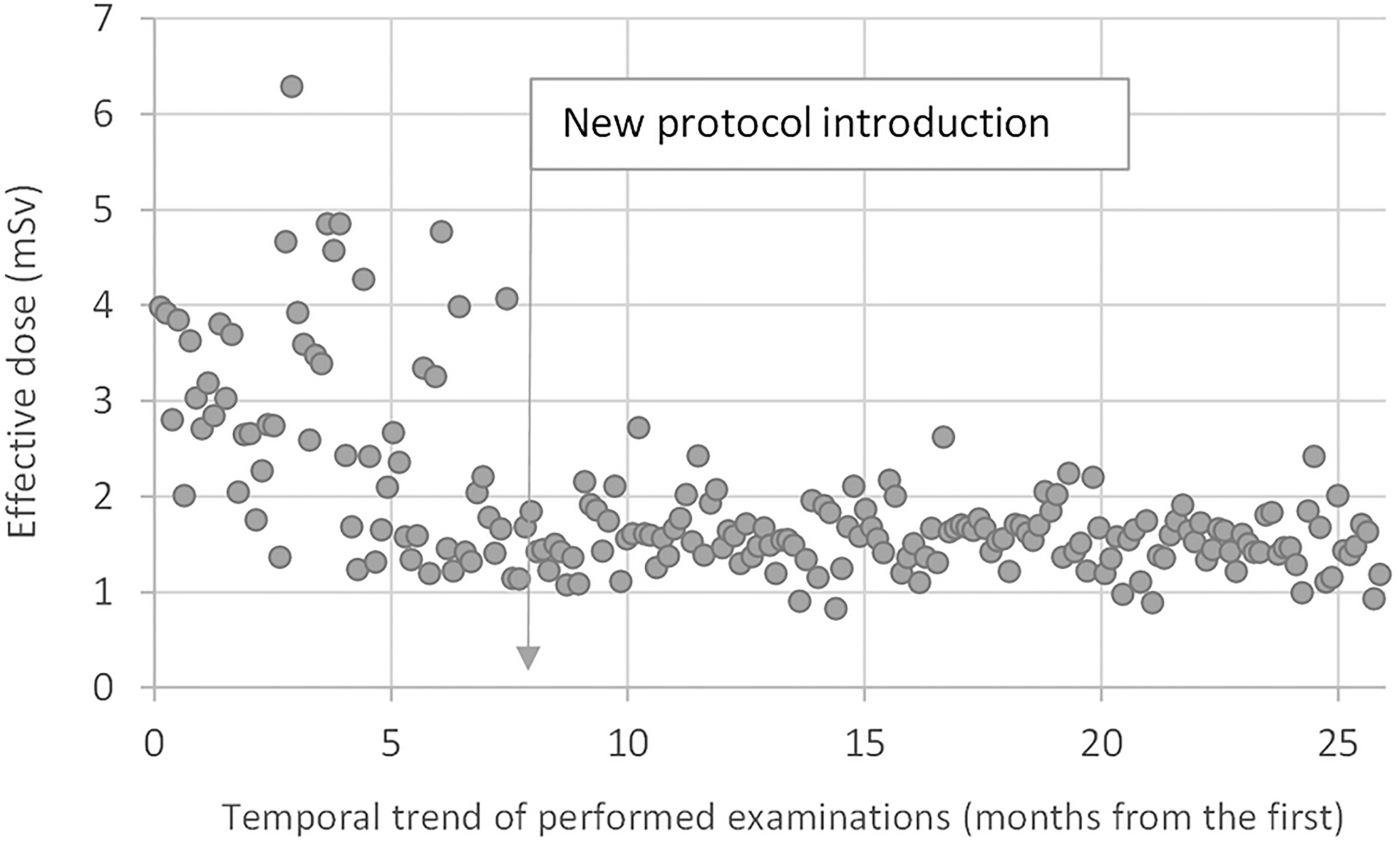
Temporal trend of the patient effective doses. The median E-value was about 2.6 mSv in the first 8 months of examinations with the standard protocol and about 1.5 mSv after the introduction of the optimized protocol.

**Figure 4** shows the temporal trend of the patient effective doses, highlighting the positive effect of the introduction of the optimized protocol. The median E value was about 2.6 mSv (IQR 1.7–3.5 mSv, mean 3.2 ± 1.1 mSv) in the first 8 months of examinations and about 1.5 mSv (IQR 1.4–1.7 mSv, mean 1.6 ± 0.5 mSv) after the new protocol introduction. Almost all values were comprised between 1 and 2 mSv.

### Image Quality

We retrospectively identified 212 patients who underwent WBLDCT for plasma cell disorder workup.

There was an overall very good agreement on image quality scores between the three readers for both groups as demonstrated by repeated measures ANOVA test with p values always superior to 0.05.

Overall image quality assessed with the same anatomic regions evaluated for the previous phantom study was very high in general and considering both groups singularly.

**Figure 5:**
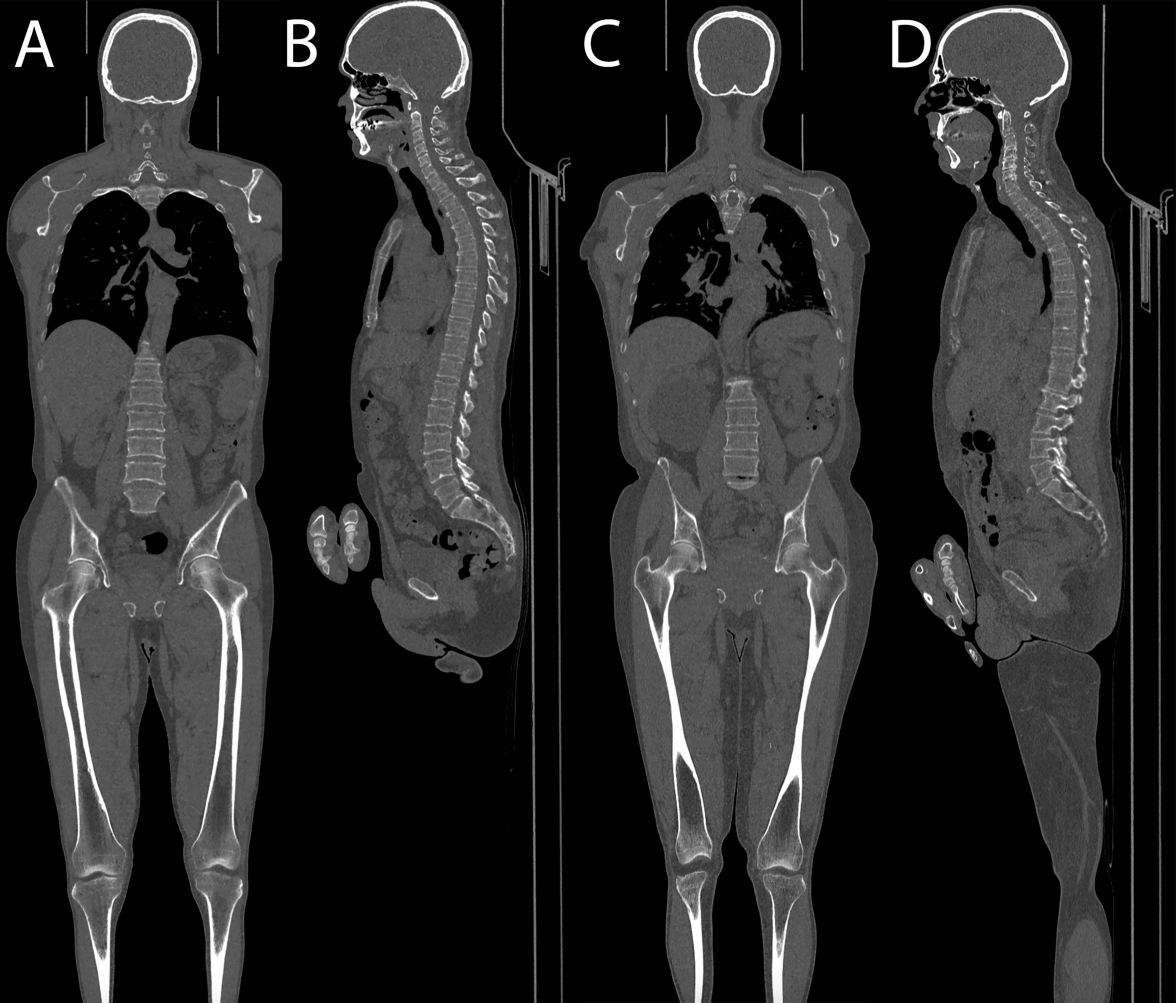
**(A, B)** Coronal and sagittal images of a patient acquired with the optimized protocol, total DLP of 157 mGy cm. **(C, D)** Coronal and sagittal images of a patient acquired with the standard protocol, total DLP of 434 mGy cm.

No statistically significant differences were demonstrated at Student’s t-test comparing mean Likert scores between standard and low-dose exams (respectively, mean score for skull, 4.94 vs. 4.93, p = 0.708; for thoracic spine, 4.88 vs. 4.89, p = 0.765; for pelvis, 4.94 vs. 4.94, p = 0.882; and for distal femora, 5 vs. 4.96, p = 0.097). Complete results are shown in **Table 2**. Sample images of a low-dose and standard protocol study are shown in **Figure 5**.

**Table 2:** Image quality scores of patients’ datasets.

Likert score of image quality of patients’ datasets												
	Group 1 standard protocol			Repeated measures ANOVA	Group 2 low-dose protocol			Repeated measures ANOVA	Standard vs. low-dose protocol group repeated measure ANOVA	Student’s t-test		
	Reader 1	Reader 2	Reader 3	p	Reader 1	Reader 2	Reader 3	p	p	Mean value standard protocol group	Mean value low-dose protocol group	p
Likert score skull base	4.88 ± 0.34	4.96 ± 0.2	5	0.174	4.93 ± 0.25	4.92 ± 0.27	4.95 ± 0.22	0.405	0.146	4.94 ± 0.231	4.9 ± 3 0.250	0.708
Likert score thoracic spine	4.88 ± 0.34	4.83 ± 0.38	4.92 ± 0.28	0.651	4.84 ± 0.38	4.91 ± 0.29	4.91 ± 0.29	0.073	0.352	4.88 ± 0.333	4.89 ± 0.322	0.765
Likert score pelvis	4.96 ± 0.2	4.96 ± 0.2	4.92 ± 0.28	0.779	4.94 ± 0.24	4.93 ± 0.26	4.95 ± 0.21	0.531	0.224	4.94 ± 0.231	4.94 ± 0.238	0.882
Likert score distal femora	5	5	5	–	4.95 ± 0.21	4.97 ± 0.16	4.96 ± 0.19	0.494	0.416	5	4.96 ± 0.189	0.097

### Osteolytic Lesions Detection

A total of 78, 79, and 78 lesions were detected by the three readers in the standard protocol group, while in the low-dose group, 360, 365, and 354 lesions were detected.

An excellent inter-rater agreement for lesion detection was achieved evaluating both groups singularly (complete results in **Table 3**) and also considering the whole patient population, obtaining high values of Fleiss’ kappa for all districts, ranging from 0.851 for humeral head to 1 for thoracic spine with values of p always <0.001.

**Table 3:** Osteolytic lesions detection inter-rater reliability.

Osteolytic lesions detection inter-rater reliability																		
	Group 1 standard protocol						Group 2 low-dose protocol group						Whole population					
	Reader 1	Reader 2	Reader 3	Agreement	Fleiss’ kappa	p	reader 1	Reader 2	Reader 3	Agreement	Fleiss’ kappa	p	Reader 1	Reader 2	Reader 3	Agreement	Fleiss’ kappa	p
Skull lesions	10	10	10	100	1	<0.001	36	35	34	98.9	0.977	<0.001	46	45	44	99.1	0.981	<0.001
Cervical spine lesions	6	6	6	100	1	<0.001	31	32	32	98.4	0.962	<0.001	37	38	38	98.6	0.968	<0.001
Thoracic spine lesions	9	9	9	100	1	<0.001	38	38	38	100	1	<0.001	47	47	47	100	1	<0.001
Lumbar spine lesions	7	7	7	100	1	<0.001	34	34	33	99.5	0.988	<0.001	41	41	40	99.5	0.99	<0.001
Humeral head	2	3	2	95.8	0.842	<0.001	10	11	10	99.5	0.966	<0.001	12	14	12	99.1	0.944	<0.001
Humeral diaphysis	5	5	5	100	1	<0.001	23	22	22	99.5	0.983	<0.001	28	27	27	99.5	0.986	<0.001
Scapular lesions	5	5	5	100	1	<0.001	25	26	26	99.5	0.985	<0.001	30	31	31	99.5	0.987	<0.001
Clavicular lesions	5	5	5	100	1	<0.001	22	20	20	98.9	0.964	<0.001	27	25	25	99.1	0.97	<0.001
Hip bone lesions	6	6	6	100	1	<0.001	40	41	41	97.9	0.958	<0.001	46	47	47	98.1	0.963	<0.001
Femoral head lesions	4	4	4	100	1	<0.001	13	15	11	96.3	0.807	<0.001	17	19	15	96.7	0.851	<0.001
Femoral diaphysis	6	6	6	100	1	<0.001	19	22	20	98.4	0.945	<0.001	25	28	26	98.6	0.957	<0.001
Sternum lesions	6	6	6	100	1	<0.001	24	22	22	97.4	0.916	<0.001	30	28	28	97.6	0.933	<0.001
Rib lesions	7	7	7	100	1	<0.001	41	42	41	98.9	0.979	<0.001	48	49	48	99.1	0.982	<0.001
Extraosseous lesion	0	0	0	100	–	–	4	5	4	99.5	0.921	<0.001	4	5	4	99.5	0.921	<0.001

**Figure 6:**
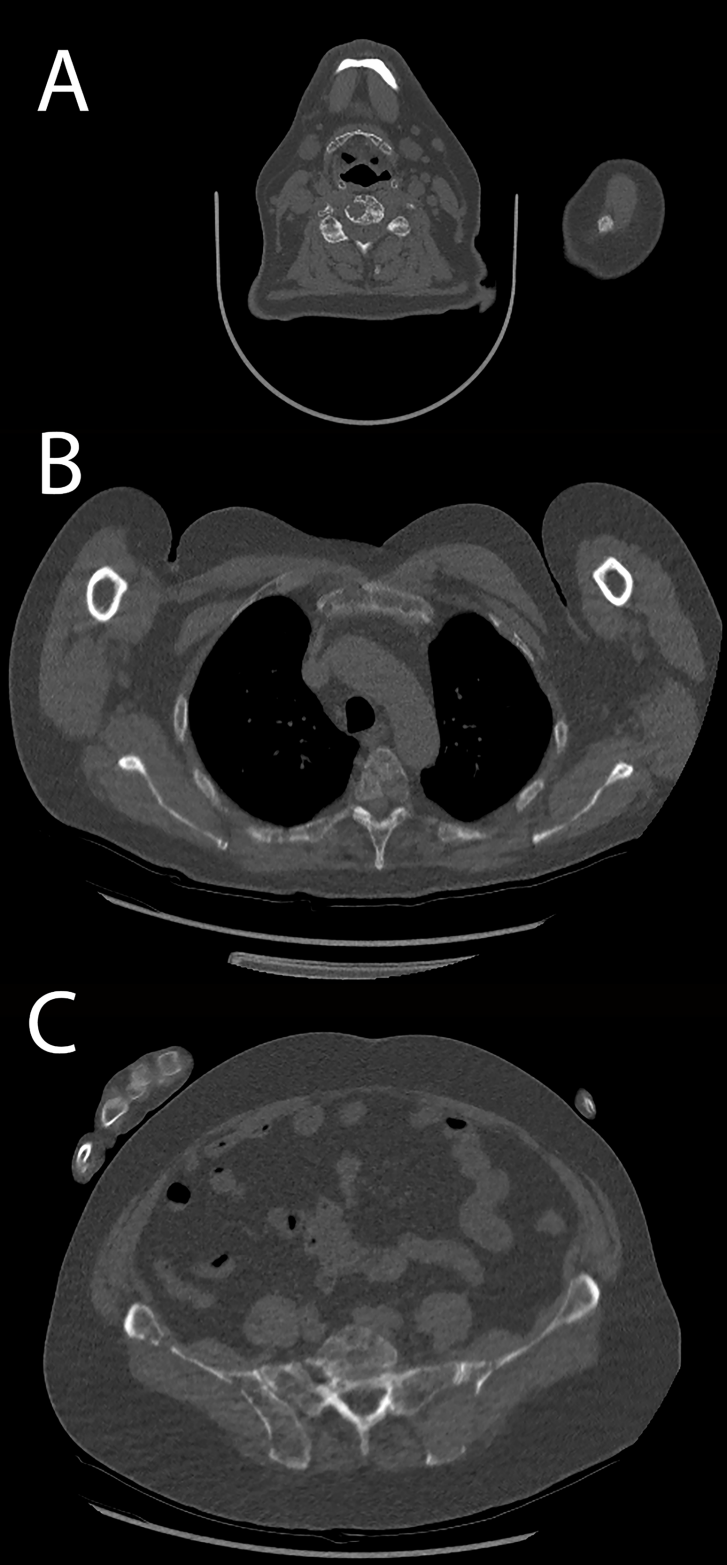
Patient acquired with standard protocol. **(A)** Cervical spine osteolytic lesions. **(B)** Thoracic spine and sternal and costal lesions. **(C)** Sacrum and hip bone lesions.

**Figure 7:**
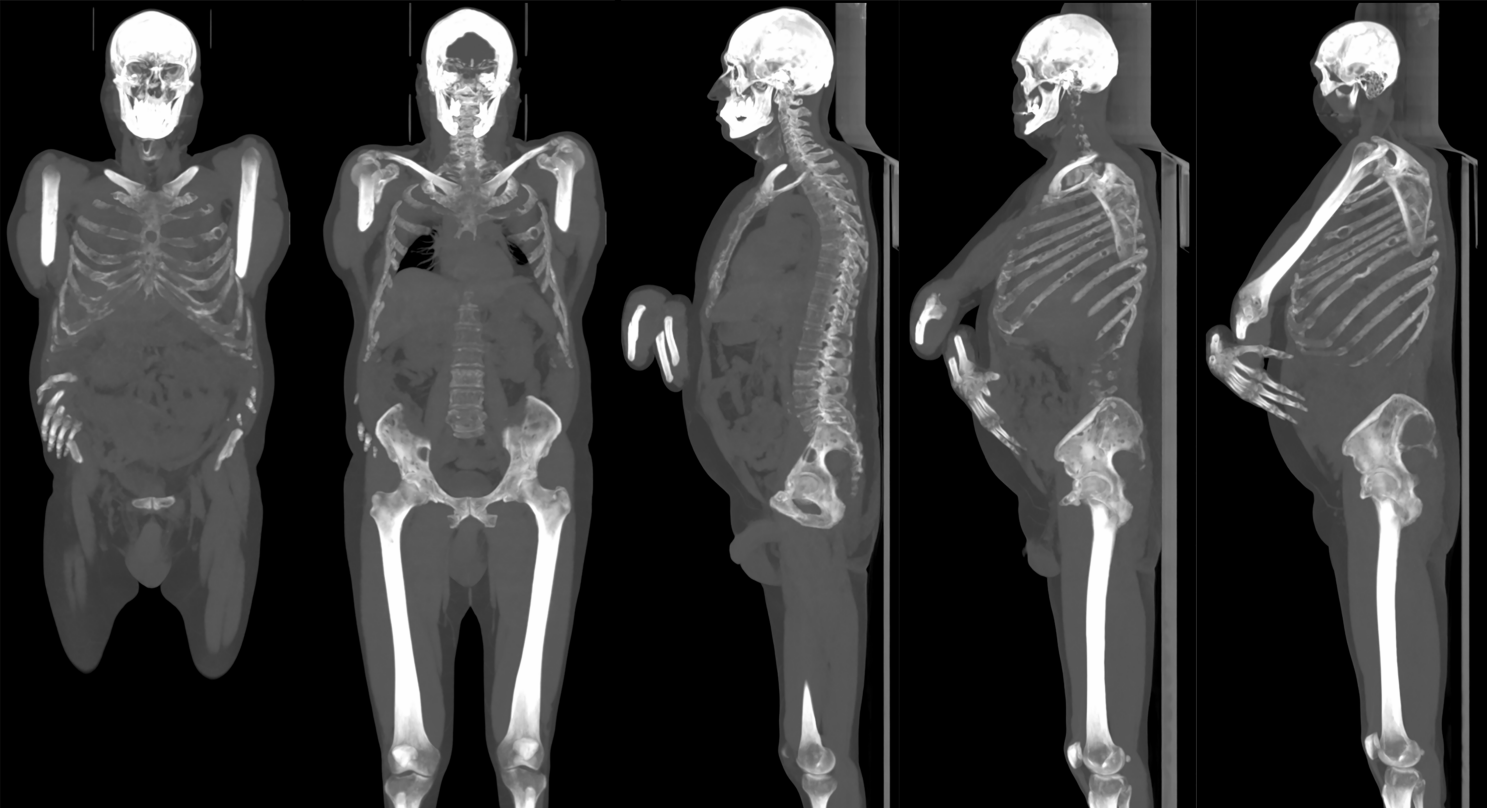
Same patient in **Figure 6**. Multiple osteolytic lesions can be appreciated on maximum intensity projection (MIP) reconstruction with thick slab (125 mm).

**Figure 8:**
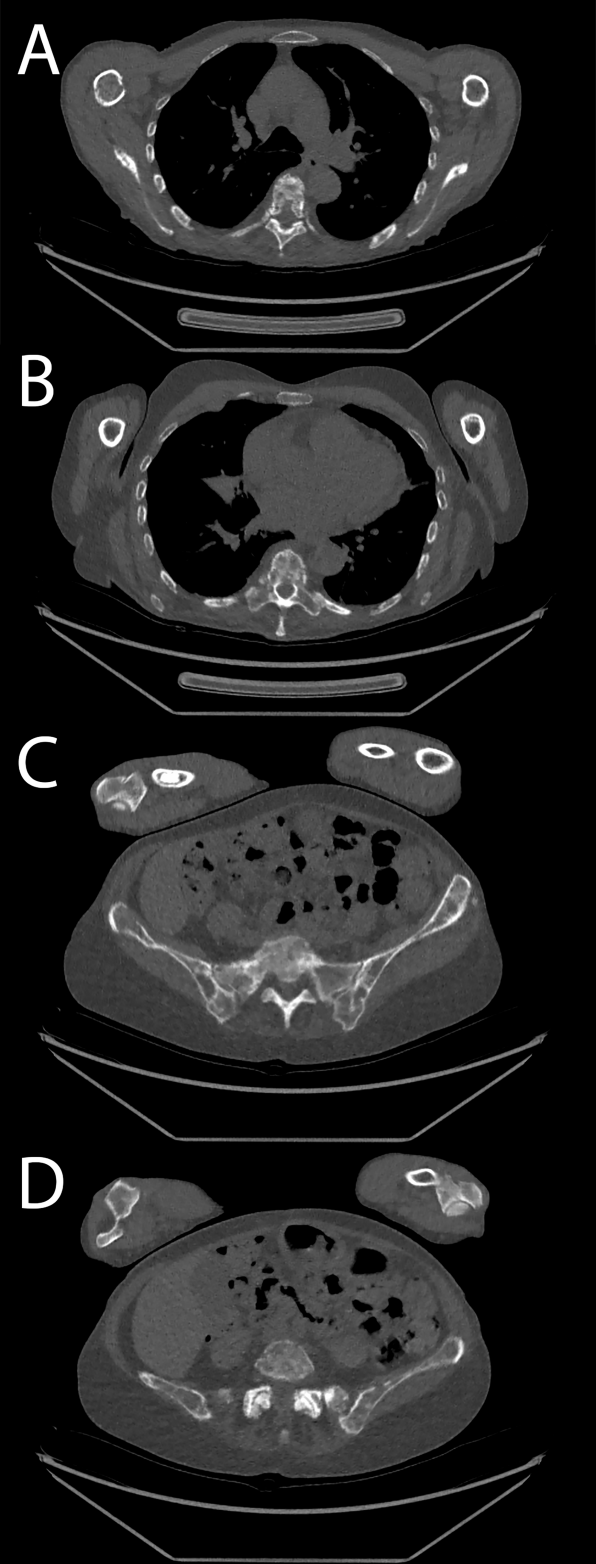
Patient acquired with low-dose protocol. **(A, B)** Thoracic spine and costal osteolytic lesion. **(C, D)** Sacrum and hip bone lesions.

A high inter-rater agreement was also found for Likert scale on diagnostic confidence for osteolytic lesions with no statistically significant differences among the readers found at Fleiss’ kappa test with p values superior to 0.05 in all districts in both groups and also considering the whole patient population (complete results in **Table 4**). Considering the mean Likert score of all readers for every single district in both groups, no statistically significant difference could be found with Student’s t-test with p-values ranging from 0.31 for femoral diaphysis lesions to 0.99 for thoracic spine lesions (complete results in **Table 5**). Sample images of osteolytic lesions are shown in **Figures 6****–****8**.

**Table 4:** Likert scores on diagnostic confidence for osteolytic lesions detection.

Likert score on diagnostic confidence for osteolytic lesions detection								
	Group 1 standard protocol			Repeated measures ANOVA	Group 2 low-dose protocol			Repeated measures ANOVA
	Reader 1	Reader 2	Reader 3	p	Reader 1	Reader 2	Reader 3	p
Skull lesions	4.8 ± 0.422	4.90 ± 0.316	5	0.368	4.94 ± 0.232	4.91 ± 0.284	4.79 ± 0.729	0.717
Cervical spine lesions	5	5	4.83 ± 0.408	0.368	4.87 ± 0.434	4.81 ± 0.397	4.75 ± 0.568	0.307
Thoracic spine lesions	4.67 ± 0.500	4.89 ± 0.333	5	0.174	4.76 ± 0.542	4.87 ± 0.343	4.89 ± 0.315	0.497
Lumbar spine lesions	5	4.82 ± 0.387	5	0.135	4.91 ± 0.379	4.82 ± 0.387	4.82 ± 0.465	0.122
Humeral head	5	5	5	0.368	4.90 ± 0.316	4.82 ± 0.405	4.7 ± 0.483	0.472
Humeral diaphysis	4.8 ± 0.447	4.2 ± 0.837	4.8 ± 0.447	0.368	4.87 ± 0.344	4.82 ± 0.395	4.91 ± 0.294	0.607
Scapular lesions	4.6 ± 0.894	4.8 ± 0.447	4.4 ± 0.894	0.497	4.83 ± 0.482	4.81 ± 0.402	4.77 ± 0.587	0.756
Clavicular lesions	5	4.6 ± 0.548	4.8 ± 0.447	0.368	4.91 ± 0.294	4.75 ± 0.444	4.85 ± 0.366	0.18
Hip bone lesions	4.83 ± 0.408	4.83 ± 0.408	4.5 ± 0.548	0.368	4.56 ± 0.788	4.46 ± 0.897	4.44 ± 0.808	0.825
Femoral head lesions	5	4.75 ± 0.500	5	0.135	4.85 ± 0.555	4.87 ± 0.352	5	0.368
Femoral diaphysis	4.67 ± 0.516	4.67 ± 0.516	5	0.264	4.95 ± 0.229	4.86 ± 0.351	4.90 ± 0.308	0.549
Sternum lesions	5	4.83 ± 0.408	5	0.135	4.96 ± 0.204	4.77 ± 0.429	4.77 ± 0.429	0.06
Rib lesions	4.86 ± 0.378	4.71 ± 0.488	4.43 ± 0.787	0.549	4.51 ± 0.746	4.57 ± 0.630	4.34 ± 0.825	0.265
Extraosseous lesion	–	–	–	–	4.71 ± 0.488	4.60 ± 0.894	4.5 ± 0.577	0.717

**Table 5:** Mean results for whole population Likert scores on diagnostic confidence for osteolytic lesions detection and comparison of mean scores for both groups.

	Mean score whole population			Repeated measures ANOVA	Student’s t for mean results of all readers		
	Reader 1	Reader 2	Reader 3	p	Mean standard protocol group	Mean low dose protocol group	p
Skull lesions	4.91 ± 0.285	4.91 ± 0.288	4.84 ± 0.645	1	4.9 ± 0.305	4.89 ± 0.466	0.924
Cervical spine lesions	4.89 ± 0.398	4.84 ± 0.370	4.76 ± 0.542	0.264	4.94 ± 0.236	4.81 ± 0.470	0.414
Thoracic spine lesions	4.74 ± 0.530	4.87 ± 0.337	4.91 ± 0.285	0.155	4.85 ± 0.362	4.84 ± 0.413	0.99
Lumbar spine lesions	4.93 ± 0.346	4.83 ± 0.381	4.85 ± 0.427	0.078	4.95 ± 0.218	4.85 ± 0.41	0.476
Humeral head	4.92 ± 0.289	4.86 ± 0.363	4.75 ± 0.452	0.472	5	4.81 ± 0.402	0.384
Humeral diaphysis	4.86 ± 0.356	4.7 ± 0.542	4.89 ± 0.32	0.264	4.6 ± 0.632	4.87 ± 0.344	0.051
Scapular lesions	4.79 ± 0.559	4.81 ± 0.402	4.71 ± 0.643	0.819	4.60 ± 0.632	4.80 ± 0.490	0.336
Clavicular lesions	4.93 ± 0.267	4.72 ± 0.458	4.84 ± 0.374	0.067	4.8 ± 0.414	4.84 ± 0.371	0.924
Hip bone lesions	4.6 ± 0.751	4.51 ± 0.856	4.45 ± 0.775	0.578	4.72 ± 0.461	4.49 ± 0.828	0.427
Femoral head lesions	4.88 ± 0.485	4.84 ± 0.375	5	0.135	4.92 ± 0.289	4.9 ± 0.384	0.984
Femoral diaphysis	4.88 ± 0.332	4.82 ± 0.390	4.92 ± 0.272	0.417	4.78 ± 0.428	4.90 ± 0.300	0.31
Sternum lesions	4.97 ± 0.183	4.79 ± 0.418	4.82 ± 0.390	0.061	4.94 ± 0.236	4.84 ± 0.371	0.441
Rib lesions	4.56 ± 0.712	4.59 ± 0.61	4.35 ± 0.812	0.607	4.67 ± 0.577	4.48 ± 0.738	0.455
Extraosseous lesion	4.71 ± 0.577	4.60 ± 0.894	4.5 ± 0.577	0.717	–	4.63 ± 0.619	–

## Discussion

The phantom study allowed us to introduce and validate a new optimized protocol for WBLDCT for patients with plasma cell disorders with a significant reduction in radiation dose compared with other experiences reported in the literature. We obtained low levels of exposure in a large cohort of patients with doses that are even lower than those reported in literature for conventional skeletal survey (ranging from 1.5 to 2.5 mSv, depending on patient BMI and equipment used) (14).

Despite a reduction of about 60% in radiation dose administered to the patients, the new lower dose protocol generated images of non-inferior diagnostic quality compared to the previous protocol, with very good or excellent quality scores. The difference in image quality scores was statistically not significant in all the anatomic districts considered.

We observed an excellent inter-rater reliability in identifying cortical osteolytic lesions in both groups considered individually and also considering the whole population with very high values of Fleiss’ kappa, thus demonstrating the non-inferiority of the low-dose protocol in lesion detection compared with the previous one.

Mean Likert scores on diagnostic confidence for lesion detection in the low-dose group ranged from 4.48 for rib and 4.49 for hip bone lesions to 4.9 for femoral head and femoral diaphysis lesions with a statistically significant difference (p < 0.001 and p = 0.003, respectively). The values of diagnostic confidence for the ribs and the hip bone regions did not present a statistically significant difference when compared to the standard protocol group (p = 0.261 and 0.246, respectively). These results are probably due to the higher noise that is present in such districts because of attenuation from surrounding structures (i.e., the spine and the arms and the hip bones and sacrum), which makes it more challenging to detect osteolytic lesions. Nevertheless, the diagnostic confidence in such regions is still very good, and it is excellent in the other districts.

Effective dose in CT is often estimated by multiplying the DLP by a proper multiplication coefficient (16). However, several recent studies pointed out the importance of considering the patient size in CT effective dose estimation, since with the same DLP, the effective dose will be significantly higher for small BMI patients and lower for large BMI patients (17–20). The use of a software with a library of virtual phantoms with different sizes allows a more accurate dose estimation. Another crucial point for an accurate E estimation for WBLDCT is the proper account of tube current modulation. We have verified that the E calculation without considering the modulation and using the average CTDI_vol_ constant over all the patients resulted in a value about 30% lower. In our study, the average ratio between the E and DLP was 0.011 mSv mGy^−1^ cm^−1^. This coefficient should be considered comparing different studies providing E-values.

Since 1985, several studies aimed at reducing radiation dose in total body CT scans for patients with plasma cell disorders. The study by Schreiman et al. demonstrated that standard-dose CT could identify more osteolytic lesions than CSS in patients with MM (5). However, using a standard-dose whole-body CT protocol resulted in unacceptable high cumulative radiation doses for patients. Mahnken et al. described a conventional high-dose CT of the thoracic and lumbar spine to evaluate bone disease in a population of patients affected by MM with effective doses ranging from 25.5 and 36.6 mSv (21). In 2005, Horger et al. published the first feasibility study for a multidetector WBLDCT protocol for MM patients, as an alternative to CSS with doses ranging from 4.1 to 7.5 mSv depending on the tube current applied with a non-significant decrease in image quality (22). Kropil et al. proposed a low-dose protocol using a tube voltage of 100 kV and an effective time–current product of 100 mAs, with automatic tube current modulation resulting in an effective dose of approximately 4.8 mSv (23). Gleeson et al. applied several different WBLDCT protocols on a single cadaver achieving a dose of 1.74 mSv, comparable to CSS while still providing sufficient diagnostic quality (24). Hemke et al. presented a retrospective study on different CT scanners with effective dose ranging from 4.34 to 8.37 mSv (25). Greffier et al. performed a phantom study where they were able to obtain images with sufficient diagnostic quality with a dose level as low as 3.4 mGy, using high levels of iterative reconstruction (26).

In our study, we obtained a median E of 1.5 mSv in a real-life population of patients, which is significantly lower than the doses reported in the works cited above. The cadaver study by Gleeson is the only other article with doses in a similar order of magnitude (24); however, a study conducted on a single human body cannot represent an accurate estimation of effective dose in a wider population with different BMIs.

Only one recent study (27) showed the possibility to perform WBLDCT with doses lower than those observed in our patient sample (average CTDI_vol_ of 0.3 mGy and DLP of 52 mGy cm), with the use of a dual source CT with spectral shaping over a population of 30 patients; however, the authors did not provide information about the patients’ BMIs, which has a considerable impact on dose estimation.

Even if most patients with MM are older adults, according to the literature, there is a non-negligible number of younger adults affected by plasma cell disorders, being about 10% of such patients younger than 50 years and 2% younger than 40 years, respectively (28). In our study population, 15 patients were 50 years old or younger (13% in group 1, 6.3% in group 2); many patients had smoldering myeloma or MGUS (respectively, 4.3% and 39.1% in group 1, 10.6% and 43.4% in group 2). Such subgroup of patients is particularly likely to undergo very long follow-ups with repeated WBLDCT scans for early identification of bone lesions (13), making it even more important to minimize radiation exposure. Dose reduction should be mandatory even in older patients, as the outcomes of plasma cell disorders are improving with current and future therapies (29); they may experience periods of disease remission or stability, and they may be treated with different lines of therapy over time. Patients with plasma cell disorders may also need to undergo exams with hybrid scanners to assess response to treatments such as PET-CT or PET-MRI, contributing to increase the radiation burden to which they are exposed.

This study has some limitations: the results cannot be directly transposed on different CT scanners, using the same acquisition parameters (i.e., kV, mA range and modulation) on a different machine, and setting a high level of iterative reconstruction may result in different performances in terms of radiation dose and image quality. For example, the minimum CTDI_vol_ achievable with tube current modulation on a GE OPTIMA CT 660 with 64-rows detector was 1.4 mGy, with greater noise and a triple total scan time (15 s) compared to the revolution CT used in this study. However, the methodology described can be applied to every CT scanner with iterative reconstruction algorithms to obtain the best compromise between radiation dose and image quality that can be achieved on different scanners. Certainly, the best performances could be expected with state-of-the-art hardware and software, but a discrete dose reduction may be achieved even on older CT scanners.

The lowest achievable dose for WBLDCT can be assessed with phantom studies and proper image quality metrics (including noise power spectrum, task transfer function, and subjective image quality scores), thus demonstrating that routine ultra-low-dose WBLDCT is feasible on latest generation CT scanners with a proper balance between tube current modulation parameters and iterative reconstruction strength, resulting in excellent image quality and diagnostic accuracy with significant dose reduction for patients respecting the low as reasonably practicable (ALARA)/as low as reasonably achievable (ALARP) principles (as low as reasonably achievable).

Technological evolution of CT scanners, the use of iterative reconstruction and adequate protocol optimization allow to obtain high-quality total body studies of patients with MM and other plasma cell disorders with radiation doses that are inferior to CSS but with an excellent sensibility in bone lesion detection.

## Data Availability Statement

The original contributions presented in the study are included in the article/supplementary material. Further inquiries can be directed to the corresponding author.

## Ethics Statement

The studies involving human participants were reviewed and approved by Comitato Etico Interaziendale A.O.U. Città della Salute e della Scienza di Torino—A.O. Ordine Mauriziano—A.S.L. Città di Torino. Written informed consent for participation was not required for this study in accordance with the national legislation and the institutional requirements.

## Author Contributions

DT and AD: conception, design, phantom study subjective image analysis, and statistical analysis. OR: conception, design, and dose analysis. CG: conception, design, and phantom study subjective image analysis. PF, RM, and SB: conception and design. MO: dose analysis and statistical analysis. ASa, ASe, and GS: *in vivo* image analysis and management of clinical data. LG: dose analysis. All authors contributed to the article and approved the submitted version.

## Conflict of Interest

The authors declare that the research was conducted in the absence of any commercial or financial relationships that could be construed as a potential conflict of interest.

## Publisher’s Note

All claims expressed in this article are solely those of the authors and do not necessarily represent those of their affiliated organizations, or those of the publisher, the editors and the reviewers. Any product that may be evaluated in this article, or claim that may be made by its manufacturer, is not guaranteed or endorsed by the publisher.
